# Effectiveness of AF pulsed field ablation with a variable-loop circular catheter: 12-month VARIPURE results

**DOI:** 10.1093/europace/euag082

**Published:** 2026-04-11

**Authors:** Daniel Scherr, Francis Bessière, Mads Brix Kronborg, Frederic Sebag, Christian Sohns, Carlo Pappone, Pedro Adragão, Tom De Potter, Sing-Chien Yap, Massimo Grimaldi, Gian Battista Chierchia, Mattias Duytschaever, Philipp Sommer, Sabine Ernst, Alexandre Almorad, VARIPURE Investigators, Carmen Rosseeuw, Wim Van Grunderbeek, Justine Zhou, Tianhui He, Tara Gomez, Yanmin Wang, Ibukun Fowe, Deborah Guest, Lycely Sepulveda-Torres

**Affiliations:** Division of Cardiology, Department of Internal Medicine, Medical University Graz, Auenbruggerplatz 15, 8036 Graz, Austria; Cardiac Arrhythmia Department, Institut de Cardiologie des Hospices Civils de Lyon—Bron, Lyon, France; Department of Cardiology, Aarhus University Hospital, Aarhus, Denmark; Unit of Cardiac Surgery and Department of Cardiology, Institute Mutualiste Montsouris, Paris, France; Department of Electrophysiology, Heart and Diabetes Center NRW, Ruhr University Bochum, Bad Oeynhausen, Germany; Arrhythmology Department, Istituto di Ricovero e Cura a Carattere Scientifico (IRCCS) Policlinico San Donato, Milan, Italy; Cardiology and Electrophysiology Department, Hospital de Santa Cruz, Lisbon, Portugal; AZORG, Cardiovascular Centre, Aalst, Belgium; Cardiology Department, Erasmus MC, Rotterdam, the Netherlands; Department of Cardiology, Ospedale Generale Regionale ‘F. Miulli’, Acquaviva delle Fonti, Bari, Italy; Heart Rhythm Management Centre, Postgraduate Program in Cardiac Electrophysiology and Pacing, Universitair Ziekenhuis Brussel—Vrije Universiteit Brussel, European Reference Networks Guard-Heart, Brussels, Belgium; Department of Cardiology, AZ Sint-Jan, Bruges, Belgium; Department of Electrophysiology, Heart and Diabetes Center NRW, Ruhr University Bochum, Bad Oeynhausen, Germany; Division of Cardiology, Royal Brompton & Harefield Hospital, London, UK; Heart Rhythm Management Centre, Postgraduate Program in Cardiac Electrophysiology and Pacing, Universitair Ziekenhuis Brussel—Vrije Universiteit Brussel, European Reference Networks Guard-Heart, Brussels, Belgium; Heart Rhythm Management Unit, Department of Cardiology, Centre Universitaire St Pierre, Université Libre de Bruxelles, Brussels, Belgium

**Keywords:** Variable-loop circular catheter, VARIPULSE, Pulsed field ablation, Atrial fibrillation, Effectiveness

Pulsed field ablation (PFA) has emerged as an effective technology for the treatment of atrial fibrillation (AF) and other cardiac arrhythmias.^1,2^ PFA with the VARIPULSE Platform [VARIPULSE variable-loop circular catheter (VLCC), TRUPULSE generator, and CARTO 3 mapping system; Biosense Webster, Inc., part of Johnson & Johnson MedTech] has been reported in registrational studies with narrow inclusion criteria, showing favourable safety and effectiveness.^3,4^ VARIPURE, a single-arm, multicentre, prospective, non-randomised, observational sub-study of the ongoing SECURE post-market follow-up study (ClinicalTrials.gov Identifier: NCT04750798), was created to address the need for evaluating the real-world safety and performance of the VLCC in contemporary clinical practice. Initial VARIPURE data have supported the ease of use, favourable safety profile, versatility, and adaptability across diverse clinical settings in patients treated in Europe.^5–7^ The aim of this article is to report interim 12-month effectiveness results of the VLCC in a real-world setting.

The study was approved by institutional ethics committees, and patients provided written informed consent prior to enrolment. Pulmonary vein isolation (PVI) was performed per standard of care, and ablation beyond the pulmonary vein regions (PVI+) was left to the operators’ discretion. After the index procedure, patients were being followed for 12 months according to routine clinical practice. Study data were acquired through an electronic data capture system and were consistently monitored, cleaned, and safety-reviewed to preserve the integrity and accuracy of the dataset. The safety endpoint was the incidence of primary adverse events (PAEs). The PAEs included the following events related to the study device and/or procedure: myocardial infarction, thromboembolism, stroke/cerebrovascular accident, transient ischaemic attack, permanent phrenic nerve paralysis, major vascular access complication/bleeding, pericarditis, or heart block within 7 days post ablation; cardiac tamponade/oesophageal perforation occurring within 30 days; and atrio-oesophageal fistula, severe pulmonary vein (PV) stenosis, or death within 12 months. Patients without ≥90 days of follow-up were excluded from the safety evaluation unless they experienced a PAE. The effectiveness endpoints were acute procedural success, defined as entrance block confirmation of all targeted PVs at the end of the procedure, and 12-month freedom from documented (symptomatic and asymptomatic) atrial arrhythmia recurrence with episodes ≥30 s recorded by electrocardiogram after a 3-month blanking period. Data were summarized descriptively; no inferential statistical tests were conducted.

From February 2024 to August 2025, 1023 patients with AF who underwent first-time ablation with the VLCC and had energy delivered [64.9 ± 10.7 years, 36.9% female, 1.9 ± 1.4 CHA_2_DS_2_-VA score, 54.8 ± 8.6% left ventricular ejection fraction, 38.8 ± 9.4 mm left atrial (LA) diameter] were enrolled across 22 European centres by 74 operators. Most patients (63.2%, 647/1023) were treated for paroxysmal AF (PAF), and 36.8% (376/1023) were treated for persistent AF (PsAF), including 41 long-standing PsAF cases. A total of 70.3% (719/1023) of patients underwent PVI-only procedures; PVI + strategies were used in 29.7% (304/1023) of patients, most commonly on the LA posterior wall [18.5% (189/1023)] or roof [13.2% (135/1023)]. The mean total procedure, pre-ablation mapping, and LA dwell times were 60.1 ± 21.8, 7.2 ± 6.5, and 38.1 ± 15.6 min, respectively. Mean fluoroscopy time was 5.2 ± 5.6 min, with 16.8% (170/1013) of cases performed without fluoroscopy. Ablation duration, valid pulsed field (PF) energy delivery time, and total number of valid PF ablations were 27.5 ± 12.0 min, 16.0 ± 4.2 s, and 22.0 ± 5.8, respectively. Acute procedural success was achieved in 99.8% (1021/1023) of patients. PAEs were observed in 0.8% (8/999) of patients who met the safety evaluation criteria, including two cardiac tamponade/perforation, four major vascular access complications/bleeding, one thromboembolism, and one pericarditis event reported (*Figure 1A*).^5^ No stroke, cerebrovascular accident, transient ischaemic attack, coronary spasm, or acute kidney injury was observed. Vagal responses were observed in 11.4% (117/1023) of cases.

**Figure 1:**
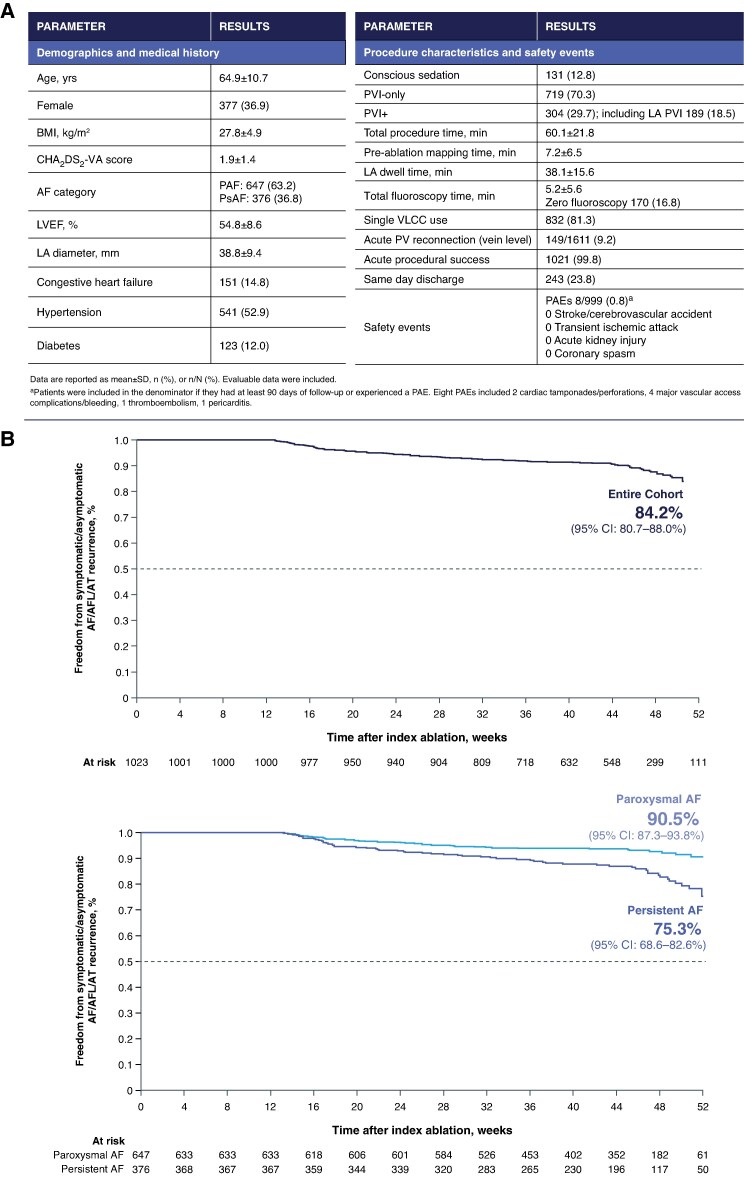
(*A*) Demographics, procedural characteristics, and safety events (*n* = 1023). (*B*) Kaplan–Meier interim analysis of 12-month freedom from atrial arrhythmia recurrence for the entire cohort (*n* = 1023), and the PAF (*n* = 647) and PsAF (*n* = 376) populations, based on available data at the time of extraction. In this interim analysis, 442 patients completed the 12-month follow-up. AF, atrial fibrillation; AFL, atrial flutter; AT, atrial tachycardia; BMI, body mass index; CHA_2_DS_2_-VA, congestive heart failure, hypertension, age ≥75 years (doubled), diabetes mellitus, stroke/transient ischaemic attack/thromboembolism (doubled), vascular disease, age 65–74 years; CI, confidence interval; LA, left atrial; LVEF, left ventricular ejection fraction; PAE, primary adverse event; PAF, paroxysmal atrial fibrillation; PsAF, persistent atrial fibrillation; PV, pulmonary vein; PVI, pulmonary vein isolation; PVI+, pulmonary vein isolation and ablations outside the pulmonary veins; SD, standard deviation; VLCC, variable-loop circular catheter.

After a median follow-up of 317.0 (interquartile range 258.0–348.0) days for the entire cohort, 43.2% (442/1023; 279 PAF, 163 PsAF) of patients completed the 12-month follow-up visit by the 17 February 2026 data extraction. Recurrences have been documented after a 3-month blanking in 16.7% (74/442; 31 PAF, 43 PsAF) of patients, including 53 and 21 cases ablated using PVI-only and PVI + strategies, respectively. Recurrent AF was the most common arrhythmia [14.3% (63/442)], followed by atrial flutter [AFL; 2.5% (11/442)], atrial tachycardia [AT; 0.7% (3/442)], and supraventricular tachycardia [0.7% (3/442)]. The cardioversion rate was 5.7% (25/442). A total of 6.3% (28/442) of patients underwent 29 repeat ablations post blanking, including 13.8% (4/29) redo procedures where all PVs remained electrically isolated. Among 72 reconnected PVs, reconnections were observed in the left inferior PV in 23.6% (17/72), left superior PV in 22.2% (16/72), right inferior PV in 26.4% (19/72), and right superior PV in 27.8% (20/72).

Data available at extraction were used to perform Kaplan–Meier analyses of treatment success for the entire cohort (*N* = 1023). A 12-month freedom from documented atrial arrhythmia (AF/AFL/AT) recurrence of 84.2% (95% CI: 80.7–88.0%) was estimated for the entire population, 90.5% (95% CI: 87.3–93.8%) for patients with PAF, and 75.3% (95% CI: 68.6–82.6%) for PsAF cases (*Figure 1B*). Likewise, freedom from documented AF recurrence was estimated at 86.8% (95% CI: 83.5–90.2%) for the entire cohort, 92.4% (95% CI: 89.8–95.1%) for PAF, and 78.9% (95% CI: 72.5–85.8%) for PsAF.

Study limitations include lack of data for on- vs. off-antiarrhythmic drug use and outcomes with different numbers of PFA applications. A comprehensive analysis of predictors of success is planned upon 12-month follow-up completion. Additionally, data for irrigation flow rates (4 mL/min or 30 mL/min) were not collected until July 2025. Therefore, relevant data before this date are unknown, and it is presumed that the default setting of 4 mL/min was used.

In this interim report from VARIPURE, the largest prospective post-market follow-up study for the VLCC to date, the results showed favourable safety profile, procedure efficiency, and 12-month efficacy in patients with AF who underwent first-time PFA with the VLCC. Although data for the entire cohort are pending, the VARIPURE 12-month effectiveness is estimated at 84.2%. The 12-month effectiveness for MANIFEST-PF, EU-PORIA, and FARADISE ranged between 74.0% and 78.1%.^8–10^ Our findings provide a strong foundation for the real-world effectiveness of the VLCC technology in routine clinical practice.

## Data Availability

Requests for study data access can be submitted through http://yoda.yale.edu.

## References

[1] Chun KJ, Miklavčič D, Vlachos K, Bordignon S, Scherr D, Jais P et al State-of-the-art pulsed field ablation for cardiac arrhythmias: ongoing evolution and future perspective. Europace 2024;26:euae134. 10.1093/europace/euae134 PMC11160504 38848447

[2] Bergonti M, Mills MT, Roten L, Ruwald MH, Metzner A, Conte G et al Pulsed field ablation for atrial fibrillation ablation: a European Heart Rhythm Association survey. Europace 2025;27:euaf294. 10.1093/europace/euaf294 PMC12686987 41363248

[3] De Potter T, Grimaldi M, Duytschaever M, Anic A, Vijgen J, Neuzil P et al Predictors of success for pulmonary vein isolation with pulsed-field ablation using a variable-loop catheter with 3D mapping integration: complete 12-month outcomes from inspIRE. Circ Arrhythm Electrophysiol 2024;17:e012667. 38655693 10.1161/CIRCEP.123.012667 PMC11111320

[4] Reddy VY, Calkins H, Mansour M, Wazni O, Di Biase L, Bahu M et al Pulsed field ablation to treat paroxysmal atrial fibrillation: safety and effectiveness in the AdmIRE pivotal trial. Circulation 2024;150:1174–86. 39258362 10.1161/CIRCULATIONAHA.124.070333 PMC11458102

[5] Almorad A, Sebag F, Kronborg MB, Sohns C, De Potter T, Adragao P et al Acute safety, effectiveness and procedural workflow for novel pulsed field ablation variable loop circular catheter in AF procedures: a prospective, multicentre, post-market clinical trial (ESC abstract). Eur Heart J 2025;46:ehaf784.796.

[6] Bessière F, Kronborg MB, Sommer P, Almorad A, Rodrigues G, Scherr D et al Evaluating safety profile and learning curve with a pulsed field ablation variable loop circular catheter in procedures for AF: observations from a prospective, multicenter, postmarket clinical trial (HRS abstract PO-07-216). Heart Rhythm 2025;22:S797. 10.1161/CIRCEP.125.014294 42158985

[7] Sommer P, Kronborg MB, Sebag F, Sohns C, De Potter T, Bessière F et al Workflow and sedation choice with the PFA variable-loop circular catheter in real-world AF procedures: insights from the prospective multi-centre VARIPURE clinical study. Europace 2026;28:euag025. 10.1093/europace/euag025 PMC12930087 41655229

[8] Boersma LVA, Széplaki G, Dello Russo A, García-Bolao I, Efremidis M, Szegedi N et al Real-world experience with the pentaspline pulsed field ablation system: one-year outcomes of the FARADISE registry. Europace 2025;27:euaf182. 10.1093/europace/euaf182 PMC12400809 40888735

[9] Schmidt B, Bordignon S, Neven K, Reichlin T, Blaauw Y, Hansen J et al EUropean real-world outcomes with Pulsed field ablatiOn in patients with symptomatic atRIAl fibrillation: lessons from the multi-centre EU-PORIA registry. Europace 2023;25:euad185. 10.1093/europace/euad185 PMC10320231 37379528

[10] Turagam MK, Neuzil P, Schmidt B, Reichlin T, Neven K, Metzner A et al Safety and effectiveness of pulsed field ablation to treat atrial fibrillation: one-year outcomes from the MANIFEST-PF registry. Circulation 2023;148:35–46. 37199171 10.1161/CIRCULATIONAHA.123.064959

